# The Interaction Effects of Meteorological Factors and Air Pollution on the Development of Acute Coronary Syndrome

**DOI:** 10.1038/srep44004

**Published:** 2017-03-09

**Authors:** Ching-Hui Huang, Heng-Cheng Lin, Chen-Dao Tsai, Hung-Kai Huang, Ie-Bin Lian, Chia-Chu Chang

**Affiliations:** 1 Division of Cardiology, Department of Internal Medicine, Changhua Christian Hospital, Changhua, Taiwan; 2 Institute of Statistics and Information Science, National Changhua University of Education, Changhua, Taiwan; 3 School of Medicine, College of Medicine, Kaohsiung Medical University, Kaohsiung, Taiwan; 4 Department of Beauty Science and Graduate Institute of Beauty Science Technology, Chienkuo Technology University, Changhua, Taiwan; 5 Division of Nephrology, Department of Internal Medicine, Changhua Christian Hospital, Changhua, Taiwan; 6 School of Medicine, Chung Shan Medical University, Taichung, Taiwan; 7 Medical Research Center, Department of Internal Medicine, Changhua Christian Hospital, Changhua, Taiwan

## Abstract

This study investigated the interaction effects of meteorological factors and air pollutants on the onset of acute coronary syndrome (ACS). Data of ACS patients were obtained from the Taiwan ACS Full Spectrum Registry and comprised 3164 patients with a definite onset date during the period October 2008 and January 2010 at 39 hospitals. Meteorological conditions and air pollutant concentrations at the 39 locations during the 488-day period were obtained. Time-lag Poisson and logistic regression were used to explore their association with ACS incidence. One-day lag atmospheric pressure (AP), humidity, particulate matter (PM_2.5_, and PM_10_), and carbon monoxide (CO) all had significant interaction effects with temperature on ACS occurrence. Days on which high temperatures (>26 °C) and low AP (<1009 hPa) occurred the previous day were associated with a greater likelihood of increased incidence of developing ACS. Typhoon Morakot was an example of high temperature with extremely low AP associated with higher ACS incidence than the daily average. Combinations of high concentrations of PM or CO with low temperatures (<21 °C) and high humidity levels with low temperatures were also associated with increased incidence of ACS. Atmospheric pollution and weather factors have synergistic effects on the incidence of ACS.

Acute coronary syndrome (ACS) is a leading cause of morbidity and mortality worldwide^1^,^2^. In Taiwan, the age-adjusted incidence of ACS (/100,000 person-years) increased from 28.0 in 1999 to 44.4 in 2008 (*p < *0.001), and the disease has been the second most common cause of mortality since 2010^3^,^4^. ACS is also a common cause of emergency hospitalization and therefore imposes a major burden on healthcare resources in that country. In Taiwan, healthcare expenditure constitutes about 6% of the gross domestic product and is expected to escalate sharply over the next two decades because of the aging population and the rise in incidence of ACS^5^. A number of risk factors for ACS have been identified, including traditional factors such as hyperlipidemia and diabetes as well as environmental factors such as weather parameters and air pollution^6^. Previous studies have indicated that weather conditions play a role in the onset of cardiovascular disease^7^,^8^. Although it is clear that weather parameters do not act alone in determining health but rather act in concert with other such parameters, e.g., ambient air pollutants and aerosols of biological origin^9^, few studies have investigated the synergistic effects of ambient air pollution and individual meteorological parameters on health outcomes.

Because of geographic and climate variations, the subject of interest varies and there is a lack of consensus about which combinations of environmental conditions are likely to contribute to the onset of ACS^10^,^11^,^12^. For example, typhoons are of particular interest in Taiwan because they occur annually, often with deleterious effects, during the months of July through September. Their impact on health such as on the incidence of ACS is of particular concern to healthcare authorities in that country. However, most studies on the impact of extreme weather on cardiovascular events have been conducted in the United States and have focused mainly on Hurricane Katrina and Hurricane Sandy^13^,^14^. Therefore, large-scale studies on the relationship between typhoons and other extreme weather events on incidence of cardiovascular events such as ACS in Asia are needed.

It is important to understand whether certain environmental factors play a role in the development of ACS. In this study we explored the relationship between meteorological parameters and ACS incidence using data on patients registered in the Taiwan ACS Full Spectrum Registry, a database established to identify predictors of outcomes of patients with ACS one year after hospital discharge at 39 medical centers and regional hospitals across Taiwan^15^. The study period investigated in this study was October 2008 to January 2010, and included the days during which typhoon Morakot (7–9 August 2009) affected Taiwan. This typhoon was the most severe in half a century and caused catastrophic damage island wide. We chose to focus on Morakot because it serves as an extreme meteorological example with which to study the relationship between ACS and environmental factors.

## Materials and Methods

### Study population and definitions

Data in this study were obtained from the Taiwan ACS Full Spectrum Registry study, a prospective, national, multicenter, non-interventional observational study on outcome of patients with ACS treated at 39 major medical centers or regional hospitals in Taiwan from 1 October 2008 to 31 January 2010 (488 days)^15^. ACS was defined in that study as symptoms ranging from ST-elevation myocardial infarction (STEMI) to unstable angina to non-STEMI. Briefly, STEMI was defined in patients who presented with acute chest pain or overwhelming shortness of breath together with persistent electrocardiographic ST elevation of >1 mm in two or more contiguous leads, or with a new or presumed new left-bundle branch block pattern on electrocardiography. Unstable angina was defined in patients who presented with acute chest pain or overwhelming shortness of breath with neither ST elevation nor abnormal levels of cardiac enzymes. Non-STEMI was defined in patients who presented with acute chest pain or overwhelming shortness of breath with no ST elevation but with a classic rise and fall in level of at least one cardiac enzyme (troponin or MB fraction of creatine kinase). To ensure that the sample satisfactorily represented the ACS population in Taiwan and that the diagnosis and management of ACS were the same at all 39 sites (including 19 tertiary medical centers and 20 regional hospitals), sites were selected by members of the Scientific Committee of the Taiwan Society of Cardiology. The accuracy of documentation was examined in 5% of case report forms at each recruiting site^16^. Blood pressure (BP) was measured in all patients with an oscillometric semiautomatic sphygmomanometer as recommended by the American Heart Association Council on High Blood Pressure Research^17^. Patient BP was measured in both arms during the initial visit and then in the arm with higher BP during subsequent visits. The recorded value was an average of two measurements. Hypertension was defined according to the 2015 guidelines of the Taiwan Society of Cardiology and Taiwan Hypertension Society for the Management of Hypertension^18^. Data on patient baseline characteristics, risk factors, clinical presentation, clinical diagnosis, in-hospital interventions, and medications prescribed were collected from admission to discharge. This study was carried out in accordance with the Declaration of Helsinki and good clinical practice. Ethics committee approval was obtained at all trial sites (list of hospitals in Appendix). Written informed consent was obtained from each patient.

### Inclusion criteria

Patients were aged 20 years or older and were admitted within 24 hours to the hospital from the emergency department with ACS symptoms. All patients provided informed consent and 3183 were eligible for inclusion and enrolled in the study. Of those patients, 3164 had a definite onset date.

### Exclusion criteria

Patients were excluded from the study if ACS was precipitated by comorbidity such as trauma, if they were previously enrolled in this trial, or if they were participating in an investigational drug study.

### Environmental data

Hourly measurements of temperature, relative humidity and air pollution (CO, NO_2_, PM_10_, PM_2.5_, SO_2_, and O_3_) were obtained from the database maintained by the Environmental Protection Administration (EPA), which has 77 monitoring stations covering most of the populated townships. Hourly measurements of atmospheric pressure (AP) were obtained from the database of the Central Weather Bureau (CWB), which has more than 700 monitoring stations throughout Taiwan. For each hospital, the three nearest monitoring stations were identified, and corresponding data of an environmental factor for that hospital were calculated as a weighted average from the three stations using reciprocals of distance to the hospital as weights. Daily data of any aforementioned environmental factor were collected and represented the average of the hourly measurements at each monitoring station. We also calculated the within-day standard deviation to represent the effect of short-term change.

### Statistical analysis

The ACS Full Spectrum data include daily numbers of ACS patients enrolled at each of the 39 medical centers during the 488-day period. For each AP-temperature stratum, the number of days and patients were summarized and means and standard deviations of corresponding measurements and proportions of comorbidities were calculated. One-way ANOVA was used to compare the demographic measurements across strata, and the chi-square test was used to compare the comorbidity proportion across strata. The relationship between number of ACS patients and measurements of meteorological factors and ambient air pollution one, two, and three days prior (lag-1, lag-2, and lag-3) to admission were explored by logistic regression. We considered 39*488 (hospital-day) observations as the binary dependent variable with value = 1 if there was at least one ACS patient for the specific hospital-day, and 0 otherwise. Logistic regression was used to fit the above binary dependent variable with various combinations of lag variables and different cutoffs for AP and temperature. Correlations of clustered data for each hospital were adjusted using a generalized estimating equation (GEE). Using the quasi-likelihood under the independence model criterion^19^ for GEE, we selected the optimal lag day and cutoffs for AP and temperature. A two-tailed *p* < 0.05 was regarded as statistically significant. All analyses were performed with the statistical package SAS for Windows (Version 9.3, SAS Institute Inc., Cary, North Carolina, USA).

## Results

### Relationship between weather parameters and air quality

Correlations between meteorological and air pollutant measurements taken one day before ACS onset in each patient are listed in Table 1. There was a significant negative correlation between AP and temperature and positive correlations between CO and NO_2_ and between PM_10_ and PM_2.5_. The aforementioned pollutants had a consistent negative correlation with temperature.

### Daily meteorological and air quality conditions and the distribution of ACS patients

Table 2 lists the number of patients with ACS in each AP-temperature category and their average age, BMI, their sex, and comorbidities (diabetes, hypertension, and dyslipidemia). There were no significant differences in these demographic and clinical characters across the six AP-temperature strata. Other clinical measurements including heart rate, BP, smoking status, family ACS history, and previous coronary artery disease are listed in Supplementary Table 1. Of those variables, only family ACS history and heart rate differed significantly across the six AP-temperature categories.

Table 3 lists the metrological and air quality information for the 488 days in the study period. Temperatures were trichotomized into low, mild and high and AP was dichotomized into low and high based on the optimal cut points determined via quasi-likelihood under the independence model criterion (QIC) as shown in Supplementary Table 2. Days with low AP and low temperatures had the highest concentrations of CO, NO_2_, PM_10_ and PM_2.5_ but the lowest O_3_ concentration. The distribution of SO_2_ was relatively uniform. The within-day standard deviations (denoted as std_wd_) of meteorological and air pollutant factors are listed in the row below the daily mean in Table 3. Most of the std_wd_ values are relatively small compared with the corresponding daily means. For example, the daily temperature ranged from 17 °C to 29 °C during the study period and the std_wd_ was roughly ±2 °C, suggesting that within-day temperature variation was modest.

As seen in Table 1, PM_10_ and PM_2.5_ could not be used simultaneously in the same model because of the strong collinearity between the two. The same is true for CO and NO_2_. After extensive trials of the combinations including interactions by examining QIC (Quasi-likelihood-based Information Criterion)^19^ and AUC (area under curve of ROC), we retained the lag-1 effect for all the factors. For air pollutants, combinations of (PM_10_ + CO) in Model (1) and (PM _2.5_ + NO_2_) in Model (2) were selected. Among the various cut-point combinations for AP-temperature that we tried, we found that [AP = 1009 hPa and temperature = 21 °C and 26 °C] and its proximities [AP = 1009 ± 1 hPa and temperature = 21 ± 1 °C and 26 ± 1 °C] had the lowest QIC and highest AUC, respectively. Table 4 lists the results from Model (1) and (2) by using cut-points [AP = 1009 hPa, temperature = 21 °C and 26 °C], and the models can be described as:

Model (1): Logit(Y) = α + β_1_ · AP_low_ + β_2_ · T_high_ + β_3_ · T_low_ + β_4_ · AP_low_ · T_high_ + β_5_ · AP_low_ · T_low_ + β_6_ · RH + β_7_RH · T_high_ + β_8_ · RH · T_low_ + β_9_ · PM_10_ + β_10_ · PM_10_ · T_high_ + β_11_ · PM_10_ · T_lw_ + β_12_ · CO + β_13_ · CO · T_high_ + β_14_ · CO · T_low_ + β_15_ · TAP_6 + _β_16_ · hospital_1_ + … + β_53_ · hospital_38_,

Model (2): Logit(Y) = α + β_1_ · AP_low_ + β_2_ · T_high_ + β_3_ · T_low_ + β_4_ · AP_low_ · T_high_ + β_5_ · AP_low_ · T_low_ + β_6_ · RH + β_7_RH · T_high_ + β_8_ · RH · T_low_ + β_9_ · PM_2.5_ + β_10_ · PM_2.5_ · T_high_ + β_11_ · PM_2.5_ · T_lw_ + β_12_ · NO_2_ + β_13_ · NO_2_ · T_high_ + β_14_ · NO_2_ · T_low_ + β_15_ · TAP_6_ + β_16_ · hospital_1_ + … + β_53_ · hospital_38_.

Here, binary Y is the indicator for ACS incidence in a hospital-day. AP_low_ = 1 for AP < 1009 hPa and = 0 otherwise. T_high_ = 1 for temperature >26 °C and = 0 otherwise, T_low_ = 1 for temperature <21 °C and = 0 otherwise. RH is relative humidity, *hospital*_*i*_ is an indicator variable for the i^th^ hospital, and the capacity difference between the 39 hospitals was considered by the offset for each hospital (β_13_–β_50_) and total ACS patients from the previous six days (TAP_6_). We did not include SO_*2*_ and *O*_*3*_ and their std_wd_ values in the model because of their insignificant contribution to the model fit. For other pollutants, we fit Model (2) by retaining all variables in Model (1), except that we replaced *PM*_*10*_ and *CO* with *PM*_*2 .5*_ and *NO*_*2*._

**Figure 1:**
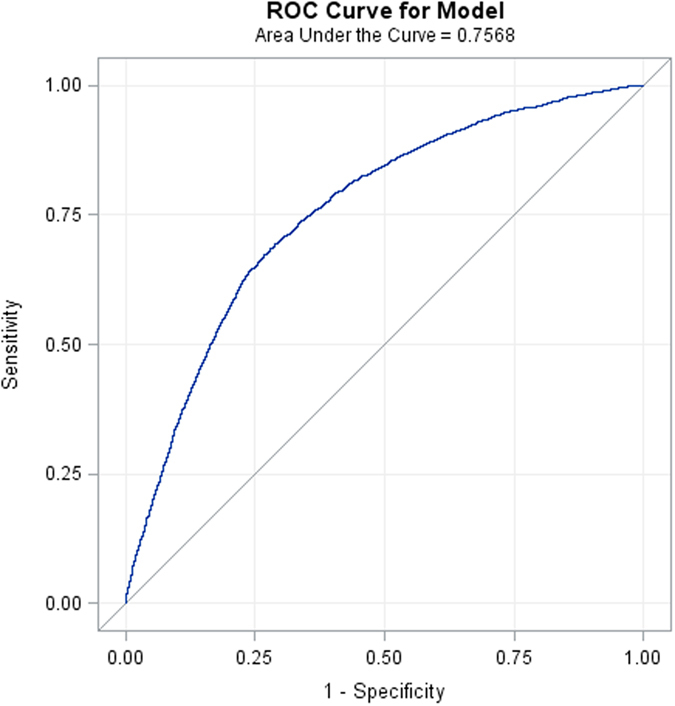
Receiver operating characteristic (ROC) curve from logistic Model (1). Area under the curve = 0.757, indicating fair model fit.

The upper part of Table 4 lists the adjusted interaction effects of temperature with AP, RH, PM_10_, and CO from Model (1), using a level of significance of 0.05. Under high temperature (>26 °C), low AP was associated with a significantly greater likelihood (OR, 2.236) of ACS onset than high AP. For other temperatures, AP variation had no significant effect. Under low temperature (<21 °C), we found that a 1% increase in RH was associated with an increased likelihood of ACS onset (OR = 1.018). For other temperatures, RH had no significant effect. Also under low temperature, we found that an increase in PM_10_ by 10 μg/m^3^ was associated with a slightly higher risk of ACS onset (OR = 1.037). Under other temperatures, PM_10_ variation had no significant effect. Also under low temperature, an increase in CO by 1 ppm was associated with significantly higher risk of ACS onset (OR = 1.122). The lower part of Table 4 lists the results for Model (2). Basically, the ORs and corresponding *p* values for AP and RH remained the same. Similar to PM_10_, an increase in PM_2.5_ concentration under low temperature was associated with a slightly higher risk of ACS (OR = 1.069). There was no significant effect under other temperatures. NO_2_ had no significant effect across all temperatures. In summary, both Model (1) and (2) suggest that on days with high temperature (>26 °C), low air pressure is a significant risk factor for ACS whereas on days with low temperature (<21 °C), high humidity and high air pollution are likely risk factors for ACS. On days with mild temperatures, however, none of the above environmental factors are significant. The receiver operating characteristic (ROC) curve for Model (1) is shown in Fig. 1. The area under the curve (AUC) for the model was roughly 0.76, indicating fair goodness of fit. The result for Model (2) was similar (data not shown).

To assess the reliability of our results, we also conducted a series analysis under the cut-point with AP = 1009 ± 1 hPa and temperature = 21 ± 1 °C and 26 ± 1 °C. The associations between ACS onset and AP-temperature remained consistent after using these cut-points.

### Daily ACS incidence and corresponding air pressure and temperature during the period of Typhoon Morakot

**Figure 2:**
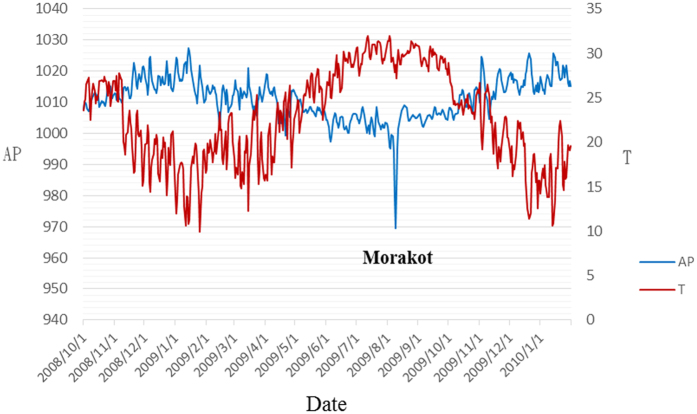
Trends in air pressure (AP) and temperature (T) during the study period.

The data for the period covering Typhoon Morakot are shown in Table 5. During the week of Typhoon Morakot, the daily incidence of ACS was higher than the study average. The time courses of AP and temperature change during the study period shown in Fig. 2 demonstrate a sudden reduction in AP during the typhoon period. The incidence of ACS was also high in the cold season with high humidity (Oct. to Dec.). Both results coincide with the summarized conclusion from Model (1) and (2).

### Daily ACS incidence and corresponding residence areas during the period of Typhoon Morakot

The most severe damage caused by Typhoon Morakot occurred in southern Taiwan. We hypothesized that people living in southern Taiwan had significantly higher levels of psychological stress after the typhoon than people living in other parts of the island. However, as shown in Table 6, there was no significant difference in incidence of ACS among the 4 geographical residence areas.

### Time course of air quality during the study period in Taiwan (1 October 2008 to 31 January 2010)

**Figure 3:**
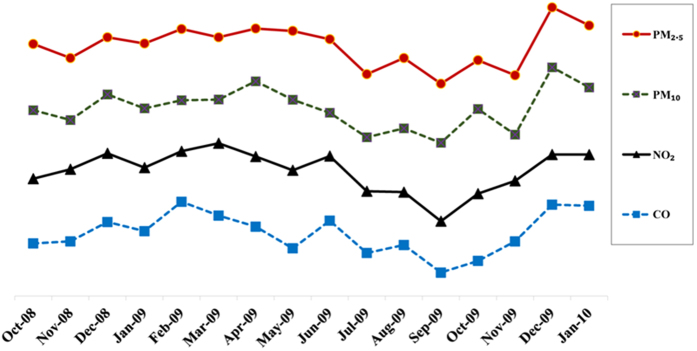
Time course of air quality during the study period in Taiwan (1 October 2008 to 31 January 2010) (standardized each pollutant).

As shown in Fig. 3, there was a downward trend in air pollution parameters after Typhoon Morakot (August 2009 to September 2009).

## Discussion

In this study we found that atmospheric pollution and weather factors have synergistic effects on the incidence of ACS in Taiwan. Previous studies have shown that temperature changes are associated with incident AMI^20^. Other studies have shown that temperature extremes or transitions/variability in temperature can trigger acute coronary events^21^,^22^. However, a systematic review on the effects of temperature on incident AMI revealed inconsistent findings^23^. Although some researchers reported that colder temperatures were associated with an increased risk of developing AMI, others reported that high temperature was a significant risk factor for AMI incidence. Possible reasons for this inconsistency may be due to geographic and climatic variations. However, another potentially important reason is that the studies included in the review used inconsistent methods^23^,^24^. Some studies adjusted for daily pollutant levels and climate parameters^25^,^26^, and some considered only temperature as a linear effect. Still others considered the possibility of lag effects of temperature in various hazard periods before AMI^27^,^28^. Furthermore, few studies were conducted in countries located in sub-tropical or tropical climates such as countries in East Asia.

The impact of ambient temperature on ACS morbidity has received attention in the past. However, temperature does not act alone in determining health, but acts in concert with other weather parameters, ambient air pollutants, and aerosol gases. The concept of “synoptic air masses” should be addressed^29^,^30^,^31^. It is reasonable to use a synoptic climatological approach to discern weather effects at a particular location and evaluate potential synergistic impacts of an entire suite of weather elements on environmental and biological parameters sensitive to weather. In our study, we determined the optimal cut points between temperature and pressure in conjunction with humidity and PM _2.5_ or PM_10_ concentrations simultaneously to explore ACS occurrence rates. We trichotomized temperature into high (>26 °C), mild (21–26 °C) and low (<21 °C) categories to address the interaction between weather parameters and ambient air pollutants. As Table 4 shows, high temperature/low AP was associated with the highest risk of developing ACS (OR = 2.236, *p* < 0.0001). In the low temperature condition, for every 1% increase in RH there was a 1.8% increase in likelihood of developing ACS (OR = 1.018, *p* < 0.0001). Also, under low temperature, for every 10 μg/m^3^ increase in PM_10_ concentration there was a 3.7% increase in likelihood of developing ACS (OR = 1.037, *p* = 0.018). Also under low temperature, for every 10 μg/m^3^ increase in PM_2.5_ concentration there was a 6.9% increase in likelihood of developing ACS (OR = 1.069, *p* = 0.032). Again under low temperature, an increase in CO by 1 ppm was associated with an increased risk of developing ACS (OR = 1.122, *p* = 0.032). This indicates that in winter, which in Taiwan is associated with high levels of humidity, a high frequency of rain and increased air pollution, the risk of developing ACS may increase. This concurs with the fact that winter has poorer air quality than other seasons. Our findings indicate that low temperature combined with high ambient CO concentration is a risk factor for developing ACS. A similar finding was reported in a study conducted in Shanghai, China^32^. CO is a well-recognized cardiovascular toxin, even at lower levels. Exposure to CO has been shown to be associated with increased risk for arrhythmias^33^, acute myocardial infarction^34^, pulmonary edema, and cardiogenic shock^35^. Allred *et al*. found that patients with coronary artery disease are more susceptible to CO-induced cardiotoxicity^36^. To the best of our knowledge, no large-scale studies have been conducted on the interaction between weather parameters and ambient air pollutants and risk for ACS in Taiwan. The strength of our study is that the confounding variables such as diabetes, hypertension, dyslipidemia, BMI, age, and sex did not have a significant effect on ACS incidence across the six AP-temperature categories (Table 2). This makes the major interaction effects of temperature, weather parameters and air pollution clearer and more precise, without adjusting for possible confounding variables.

Ambient air particles are a complex mixture of inorganic and organic compounds originating from different sources^37^, each with complex particle size distributions. An increasing number of studies suggest that ultrafine particles (aerodynamic diameter smaller than approximately 0.1–0.2 μm) as compared with larger particles, which can dominate PM_2.5_ mass, may have greater adverse cardiovascular effects because of higher deposition efficiency and larger surface area^38^, as well as higher redox activity^39^. The association between short-term exposure to particulate air pollution and risk for myocardial infarction has been well studied. Studies have shown that short-term exposure to particulate matter (PM_2.5_, PM_10_) is a risk factor for MI and that the risk associated with exposure to PM_2.5_ is greater than the risk associated with exposure to PM_10_^40^. However, ambient PM mass measurements may not adequately represent risk for cardiovascular disease because they are uncharacterized by composition, source or oxidative potential^41^. A recent study demonstrated that the triggering of myocardial infarction by fine particles is enhanced when particles are enriched in secondary species (sulfate, nitrate, and/or organics)^42^. Previous experimental results indicated that the mean fine particulate and coarse particulate concentrations are higher in spring and winter than in summer and autumn in Taiwan^43^. Those results confirm our finding that air pollution is more severe in winter. Lee *et al*. showed that seasonal variation in composition of PM could be judged by atmospheric visibility^44^. They found that the seasonal mean visibilities in spring, summer, fall, and winter were only 5.4, 9.1, 8.2, and 3.4 km, respectively. Sulfate was a dominant component that affected both the light scattering coefficient and the visibility in Kaohsiung City, the largest urban city in southern Taiwan. On average, (NH_4_)_2_SO_4_, NH_4_NO_3_, total carbon, and fine particulate matter (PM_2.5_) contributed 53%, 17%, 16%, and 14% to total light scattering, respectively.

Another interesting finding in our study was the observation that the incidence of ACS was higher on the day of and on subsequent days after typhoon Morakot. On 5 August 2009, tropical storm Morakot developed into a typhoon and AP decreased steadily as the typhoon strengthened. The typhoon reached peak intensity early on 7 August with a minimum pressure of 945 hPa and sustained winds of 161 km/h (100 mph), equivalent to a Category 2 hurricane. Later that day, the typhoon made landfall in central Taiwan. About 24 hours later on 9 August, the storm moved off the west coast of the island into the Taiwan Strait, where it turned north-northwest. As seen in Fig. 2, the typhoon was an extreme example of high temperature/low AP, which concurred with our previous statement that high temperature combined with low atmospheric pressure is associated with the highest likelihood of developing myocardial infarction. In addition, this typhoon was a natural disaster, causing severe damage in Taiwan. Table 5 shows that in the typhoon period (7–12 August), the number of ACS cases was greater than average across the entire island. Previous studies have shown an increase in AMI during the immediate hours to weeks following natural disasters^45^. However, most studies have been conducted on the long-term effects of extreme weather events, such as Hurricane Katrina, which have been shown to be associated with an increased incidence of ACS occurring for up to six years after the hurricane^13^,^46^,^47^. A single-center study found a 3-fold increase in the proportion of admissions for AMI in the 2 years following Katrina (2.18% vs. 0.71%, p < 0.0001), and also noted that the post-Katrina group had higher rates of unemployment, lack of insurance, medication non-compliance, smoking, and substance abuse^13^. The short-term effect of extreme weather on ACS morbidity has also been investigated. For example, one study showed that 2 weeks following Hurricane Sandy, there was a marked increase in the incidence of myocardial infarction^14^. Furthermore, another study on Hurricane Sandy also demonstrated that diabetic adults aged 65 years and older were especially at risk for developing acute myocardial infarction the week after that extreme weather event^48^. The development of ACS in the above-mentioned studies was attributed to the psychological stress of the event and corresponding physiologic derangements^49^. Although we agree that acute psychological stress plays a role in triggering cardiac events, our results show that other factors are also involved. For example, the most severe damage caused by Typhoon Morakot occurred in southern Taiwan. It is reasonable to hypothesize that people living in southern Taiwan had significantly higher levels of psychological stress after the typhoon than people living in other parts of the island. Therefore, we analyzed the daily ACS incidence during the typhoon period according to corresponding residence areas and tried to find whether the ACS incidence was higher in southern Taiwan. However, there was no significant difference in incidence of ACS among the 4 geographical residence areas (Table 6). This finding implies that the increase in ACS incidence after exposure to extreme weather could not be explained by psychological stress alone. Our data suggest that changes in environmental factors combined with extreme weather may have triggered AMI development. Typhoons are predictable but still destructive. Although we cannot prevent natural disasters, it is feasible to organize ACS teams to cope with the potential for increased ACS patient care needs^50^.

There are other environmental factors that have been shown to be associated with increases in incidence of MI besides extreme weather events. Typhoons are frequently accompanied by high wind speeds and unstable weather conditions that also dilute and eliminate air pollutants^51^. One study conducted in Hsinchu Science Park in Taiwan demonstrated that the concentration of polar compounds dropped sharply from 41.4 ppbv before the typhoon to the stage of no detection during the storm, but rose again after the typhoon^52^. Our findings are also similar to those reported by Swerdel *et al*. on Hurricane Sandy in New Jersey^14^. In that study, the authors examined the levels of ambient fine particulate pollution (PM_2.5_) in New Jersey 2 weeks before and 2 weeks after Hurricane Sandy made landfall on October 29, 2012. They found that the levels of this pollutant were slightly lower in the 2 weeks following Sandy than in the preceding 2 weeks. Our study revealed a downward trend in air pollution during the typhoon period (Fig. 3). Another important environmental factor is influenza. Influenza outbreaks have been reported to be associated with an increase in incidence of MI^53^. During the days following typhoon Morakot in August 2009, there was no significant increase in influenza cases. In Taiwan, influenza outbreaks mainly occur from late November to March^54^. The aforementioned statements suggest that these two important environmental factors (influenza and air pollution) did not play a significant role in the incidence of ACS following Typhoon Morakot.

Our findings are similar to those reported by Houck *et al*. who found that rapid AP decreases are associated with the occurrence of AMI but not with that of stroke^55^. Although the mechanism is unclear, we hypothesize that changes in AP might contribute to plaque rupture. However, the relationship between AP and ACS risk is inconsistent because of geographic and climate variations and inconsistent methods.

There are some limitations to our study. First, we used data from EPA and CWB monitoring stations near the hospitals rather than obtaining data on exposure to environmental factors for each patient. Second, some cases may not have been enrolled in the registry databank. Third, we did not provide laboratory parameters or functional assessment data, which may have major impacts on ACS mortality. Fourth, we did not have data on living habits such as exercise and diet, which may confound our conclusions. Fifth, we did not assess PM composition or micro-pollutant exposure data in our study, so we cannot provide information on the lag effect of chemical components. Longer observation times may be needed in the future.

## Conclusions

One-day lag AP, RH, PM_2.5_ and PM_10_ all had significant interaction effects with temperature on the occurrence of ACS. Days on which high temperatures (>26 °C) and low AP (<1009 hPa) occurred the previous day were associated with a greater likelihood of increased incidence of developing ACS. Typhoon Morakot was an example of high temperature with extremely low AP associated with higher ACS incidence than the daily average. These findings, however, should be interpreted with caution and longer observation periods are needed in the future. Combinations of higher PM_2.5_, PM_10_ or CO concentrations with lower temperature, and higher RH with lower temperature, were also associated with ACS occurrence. Better understanding of these meteorological patterns and air quality interactions may provide additional measures for cardiovascular disease prevention.

## Additional Information

**How to cite this article:** Huang, C.-H. *et al*. The Interaction Effects of Meteorological Factors and Air Pollution on the Development of Acute Coronary Syndrome. *Sci. Rep.* **7**, 44004; doi: 10.1038/srep44004 (2017).

**Publisher's note:** Springer Nature remains neutral with regard to jurisdictional claims in published maps and institutional affiliations.

## Supplementary Material

Supplementary Information

## Figures and Tables

**Table 1:** Pearson coefficients of correlation between meteorological and air pollutant daily measurements during the 488-day study period.

	AP*	T	RH	CO	NO_2_	PM_2.5_	PM_10_	SO_2_	O_3_
AP	1								
T	**−0**.78*	1							
RH	−0.24	0.12	1						
CO	0.37	−0.34	0.06	1					
NO2	0.41	−0.41	−0.07	**0**.89*	1				
PM2.5	0.22	−0.17	−0.2	0.38	0.5	1			
PM10	0.25	−0.2	−0.26	0.28	0.41	**0**.88*	1		
SO2	0.14	0.04	−0.14	0.34	0.46	0.66	0.61	1	
O3	−0.06	0.16	−0.35	−0.14	−0.06	0.4	0.37	0.15	1

AP: air pressure, T: temperature, RH: relative humidity.

**Table 2:** Distribution of ACS patients for 488-day study period *39 study hospitals, stratified by air pressure (AP) and temperature (T).

	high T (>26 °C)		mild T (21–26 °C)		low T (<21 °C)		p value!
	low AP (<1009 hpa)	high AP (≥1009 hpa)	low AP	high AP	low AP	high AP	
Number of hospital days	6288	748	2274	3021	164	6537	‒
Total case no.	1306	54	380	489	16	919	
Case per hospital day	0.21	0.07	0.17	0.16	0.1	0.14	<0.001
Gender (male %)	1018/1306 (78.0%)	42/54 (77.8%)	300/380 (79.0%)	364/489 (74.4%)	15/16 (93.8%)	727/919 (79.1%)	0.238
Age	62.55 ± 13.44	64.31 ± 14.19	63.43 ± 14.1	63.22 ± 13.67	65.19 ± 15.63	63.29 ± 13.36	0.652
BMI	25.45 ± 3.84	24.7 ± 3.49	25.3 ± 4.08	25.4 ± 3.92	26.09 ± 6.09	25.44 ± 3.92	0.776
Diabetes no.	459 (35.4%)	16 (29.6%)	117 (30.8%)	195 (40.1%)	7 (43.75%)	334 (36.7%)	0.08
Dyslipidemia no.	512 (39.7%)	17 (31.5%)	155 (40.8%)	190 (39.1%)	7 (43.75%)	344 (37.8%)	0.739
Hypertension no.	796 (61.7%)	36 (67.9%)	254 (66.8%)	312 (64.3%)	12 (75%)	591 (65.1%)	0.3

^*^: ±one standard deviation.

!: Across-strata comparison: one-way ANOVA for age and BMI, and chi-square test for gender and comorbidity proportions.

**Table 3:** Mean and within-day standard deviation (std_wd_) of meteorological and air pollutant factors, averaged over specific hospital-days stratified by temperature (T) and air pressure (AP).

		high T (>26 °C)		mild T (21–26 °C)		low T (<21 °C)		p value!
		low AP (<1009 hpa)	high AP (≥1009 hpa)	low AP	high AP	low AP	high AP	
Number of hospital-days		6288	748	2274	3021	164	6537	‒
RH	Mean	73.54 ± 6.47	75.61 ± 4.55	73.47 ± 10.18	72.97 ± 8.21	79.05 ± 6.38	72.2 ± 9.89	<0.001
	std_wd_	8.71	8.78	8.8	8.94	7.23	8.21	
CO	Mean	0.48 ± 0.24	0.6 ± 0.25	0.58 ± 0.27	0.62 ± 0.26	0.57 ± 0.26	0.65 ± 0.46	<0.001
	std_wd_	0.25	0.29	0.27	0.3	0.22	0.3	
NO2	Mean	15.92 ± 7.2	19.08 ± 7.62	20.51 ± 8.27	22.19 ± 8.48	20.18 ± 8.58	22.12 ± 8.2	<0.001
	std_wd_	7.06	8.07	8.79	9.25	8.68	9.08	
PM10	Mean	50.06 ± 22.44	71.48 ± 31.3	64.24 ± 30.96	72.51 ± 41.43	56.19 ± 33.36	66.69 ± 34.72	<0.001
	std_wd_	14.81	21.48	22.74	22.41	20.24	22.17	
PM2.5	Mean	29.57 ± 13.9	41.18 ± 19.25	37.08 ± 17.14	40.28 ± 21.51	35.1 ± 22.29	37.19 ± 18.62	<0.001
	std_wd_	9.51	12.64	12.55	12.55	12.96	12.15	
SO2	Mean	4.25 ± 2.07	5.16 ± 2.32	3.92 ± 1.91	4.58 ± 2.6	3.03 ± 1.48	4.12 ± 2.13	<0.001
	std_wd_	2.57	2.99	2.06	2.44	1.56	1.96	
O3	Mean	27.88 ± 12.71	33.54 ± 11.16	33.6 ± 13.08	32.02 ± 10.67	27.48 ± 8.53	25.92 ± 9.09	<0.001
	std_wd_	16.95	21.56	17.12	17.98	13.07	13.16	
T	Mean	28.87 ± 1.46	27.24 ± 0.8	24.34 ± 1.26	23.04 ± 1.45	19.53 ± 1.13	17.59 ± 2.45	<0.001
	std_wd_	1.97	2	2.09	2.14	1.69	2.01	

The std_wd_ was estimated by the hourly measurement of each day.

!: Across-strata comparison: one-way ANOVA for age and BMI, and chi-square test for gender and comorbidity proportions.

**Table 4:** Interactions of temperature with air pressure (AP), relative humidity (RH) and air pollutants for ACS: odds ratio (OR) of AP, RH, PM_10_ and CO (from Model (1)), and PM_2.5_ and NO_2_ (from Model (2)) in different temperature strata, after adjusting the offsets by hospital.

	high T (>26 °C)		mild T (21–26 °C)		low T (<21 °C)	
	OR* (95% CI)	p-value	OR (95% CI)	p-value	OR (95% CI)	p-value
***Model*** (***1***)						
Low AP: High AP	**2**.**236** (**1**.**572**, **3**.**181**)	**<0**.**0001**	1.124 (0.899, 1.405)	0.305	0.811 (0.434, 1.517)	0.513
RH per 1%^↑^	0.995 (0.982, 1.009)	0.501	0.997 (0.987, 1.005)	0.38	**1**.**018** (**1**.**009**, **1**.**027**)	**<0**.**0001**
PM10 per 10 μg/m^3↑^	0.983 (0.948, 1.019)	0.352	1.006 (0.983, 1.029)	0.601	**1**.**037** (**1**.**006**, **1**.**067**)	**0**.**018**
CO per 1 ppm^↑^	0.822 (0.568, 1.191)	0.301	0.952 (0.648, 1.398)	0.8	**1**.**122** (**1**.**010**, **1**.**246**)	**0**.**032**
***Model*** (***2***)						
Low AP: High AP	**2**.**193** (**1**.**54**, **3**.**124**)	**<0**.**0001**	1.116 (0.894, 1.392)	0.334	0.793 (0.423, 1.488)	0.471
RH per 1%^↑^	0.993 (0.979, 1.008)	0.356	0.997 (0.986, 1.005)	0.318	**1**.**018** (**1**.**009**, **1**.**026**)	**<0**.**0001**
PM2.5 per 10 μg/m^3↑^	0.961 (0.91, 1.015)	0.152	0.980 (0.928, 1.035)	0.459	**1**.**069** (**1**.**006**, **1**.**137**)	**0**.**032**
NO2 per 10 ppb^↑^	0.90 (0.801, 1.003)	0.06	0.989 (0.861, 1.136)	0.874	0.984 (0.866, 1.118)	0.805

**Table 5:** Daily ACS incidences and corresponding air pressure and temperature before and after landfall of Typhoon Morakot (7–13 August 2009).

Date	Mar	April	May	June	July	8/7	8/8	8/9	8/10	8/11	8/12	8/13	Sep	Oct	Nov	Dec	Jan
ACS	5.1	6.6	8	9	8.29	10	9	7	10	9	7	12	8.57	9.48	10.17	10.9	8.7
AP*	1011	1008.9	1007.2	1002.2	1002.7	992.9	977.3	971.5	983.8	995.6	1001.9	1004.1	1003.7	1007.4	1012.5	1015	1016.1
T	20.1	22.3	26	28.2	29.8	28.32	27.83	27.68	27.22	28.23	29.71	28.8	29.5	25.5	22.5	18.1	17.3

^*^The overall average of 488 days is 6.48 case/day (95% CI: [6.09, 6.87]), with mean AP 1009.5 (95% CI: [1008.9, 1010.1]) and temperature 23.39 (95% CI: 22.95, 23.82]). For the “hot” week around the impact of Morakot (7–13 August), there were 9.14 incidences per day.

**Table 6:**
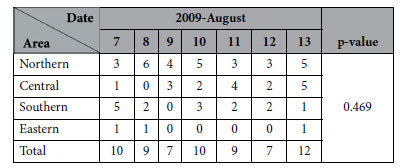
Daily ACS incidence and corresponding geographic residence areas during the period of Typhoon Morakot.

We used the chi-square test for independence to determine the correlation between ACS incidence and region.
