# Supplementary material for: The Interaction Effects of Meteorological Factors and Air Pollution on the Development of Acute Coronary Syndrome
Source: Sci Rep. 2017 Mar 9;7:44004. doi: 10.1038/srep44004 (PMC5343658; doi:10.1038/srep44004)
Supplement: Supplementary Information [file srep44004-s1.pdf]

The Interaction Effects of Meteorological Factors and Air Pollution on the Development of Acute Coronary Syndrome

Ching-Hui Huang1, 2, 3, 4, Heng-Cheng Lin2, Chen-Dao Tsai1, Hung-Kai Huang1, Ie-Bin Lian2, *, Chia-Chu Chang5, 6, 7, *

1. Division of Cardiology, Department of Internal Medicine, Changhua Christian Hospital, Changhua, Taiwan.

2. Institute of Statistics and Information Science, National Changhua University of Education, Changhua, Taiwan.

3. School of Medicine, College of Medicine, Kaohsiung Medical University, Kaohsiung, Taiwan.

4. Department of Beauty Science and Graduate Institute of Beauty Science Technology, Chienkuo Technology University, Changhua, Taiwan.

5. Division of Nephrology, Department of Internal Medicine, Changhua Christian Hospital, Changhua, Taiwan.

6. School of Medicine, Chung Shan Medical University, Taichung, Taiwan.

7.Medical Research Center, Department of Internal Medicine, Changhua Christian Hospital, Changhua, Taiwan.

Supplementary Table 1: Daily meteorological and air quality conditions and distribution of ACS patients

	high T (>26°C)		mild T (21–26°C)		low T (<21°C)		
	low AP(<1009hpb)	high AP(?1009hpb)	low AP	high AP	low AP	high AP	p-value
Heart Rate(beat/min)	81.44±21.49	78.96±23.13	84.22±23.88	83.77±21.59	93.25±39.81	81.91±23.23	0.037
Blood Pressure							
Systolic	137.73±31.29	136.54±31.03	143.08±36.53	139.28±31.87	134.31±30.6	140.42±33.82	0.084
Diastolic	81.5±20.65	77.17±18.18	83.79±21.32	81.55±20.68	79.81±19.23	81.2±21.32	0.225
Smoker							
Current	537/1280(41.95%)	27/53(50.94%)	172/372(46.24%)	196/487(40.25%)	5/14(35.71%)	369/903(40.86%)	0.551
Former	222/1280(17.34%)	6/53(11.32%)	53/372(14.25%)	80/487(16.43%)	4/14(28.57%)	161/903(17.83%)	
Never	521/1280(40.7%)	20/53(37.74%)	147/372(39.52%)	211/487(43.33%)	5/14(35.71%)	373/903(41.31%)	
Family History	215/929(23.14%)	8/46(17.39%)	73/293(24.91%)	80/385(20.78%)	3/11(27.27%)	159/718(22.14%)	0.004
Known CAD	299/1306(22.89%)	13/54(24.07%)	96/380(25.26%)	129/489(26.38%)	2/16(12.5%)	234/919(25.46%)	0.468
Previous MI	124/292(42.47%)	4/13(30.77%)	39/92(42.39%)	52/127(40.94%)	0/2(0%)	93/231(40.26%)	0.803
Previous PCI	205/296(69.26%)	8/13(61.54%)	66/91(72.53%)	79/126(62.7%)	2/2(100%)	168/233(72.1%)	0.398
Previous CABG	37/294(12.59%)	1/13(7.69%)	5/91(5.49%)	15/128(11.72%)	0/2(0%)	28/231(12.12%)	0.53

Supplementary Table 2: Optimal cut points for temperature (T) and air pressure (AP) determined by quasi-likelihood under the independence model criterion (QIC) of logistic regression

Cut point			QIC	AUC	Days of high t high AP	Days of low t low AP
AP	Low T	High T				
1010	22	27	13446.58*	0.758	270	643
1010	21	27	13452.29	0.7576	270	388
1010	20	27	13461.06	0.7567	270	249
1010	23	27	13448.54	0.7576	270	969
1010	24	27	13450.43	0.7574	270	1452
1010	22	26	13465.56	0.7578	471	643
1009	22	26	13480.33	0.757	748	325
1010	22	25	13470.52	0.7571	778	643
1010	21	26	13470.74	0.7575	471	388
1009	21	26	13483.23	0.7568	748	164
1011	21	26	13501.01	0.7556	238	865
1011	22	27	13480.45	0.7561	120	1233
1009	22	27	13474.38	0.757	425	325
1009	23	27	13473.39	0.7569	425	550

Supplementary Table 3. Interactions of temperature with air pressure (AP), relative humidity (RH) and air pollutants for ACS: odds ratio (OR) of AP, RH, PM_{10} and CO (from Model (1)), and $PM_{2.5}$ and NO_2 (from Model (2)) in different temperature strata, after adjusting the offsets by hospital (1010 hPa, 22 and 26 °C)

Model (1)	High temperature		Mild temperature		Low temperature	
	OR* (95%CI)	p-value	OR (95%CI)	p-value	OR (95%CI)	p-value
Low AP : High AP	**3.868 (1.844,8.116)**	**0.0003**	1.165 (0.940,1.443)	0.164	0.751 (0.540,1.045)	0.089
RH per 1 % ↑	0.994 (0.981,1.008)	0.395	0.993 (0.940,1.443)	0.2	**1.018 (1.009,1.027)**	**0.0001**
PM10 per 10μg/m3 ↑	0.974 (0.940,0.010)	0.16	1.003 (0.977,1.029)	0.808	**1.032 (1.008,1.057)**	**0.009**
CO per 1 ppm ↑	0.903 (0.625,1.304)	0.585	1.081 (0.771,1.516)	0.652	**1.124 (1.014,1.246)**	**0.026**
Model (2)	High temperature		Mild temperature		Low temperature	
	OR*(95%CI)	p-value	OR(95%CI)	p-value	OR(95%CI)	p-value
Low AP : High AP	**3.774 (1.803,7.902)**	**0.0004**	1.164 (0.944,1.435)	0.155	0.750 (0.537,1047)	0.091
RH per 1% ↑	0.993 (0.979,1.007)	0.319	0.994 (0.983,1.004)	0.217	**1.018 (1.009,1.027)**	**<.0001**
PM2.5 per 10μg/m3 ↑	0.955 (0.903,1.009)	0.098	0.955 (0.902,1.011)	0.111	**1.074 (1.019,1.133)**	**0.008**
NO2 per 10 ppb ↑	0.907 (0.810,1.017)	0.095	1.047 (0.916,1.196)	0.501	0.958 (0.848,1.082)	0.491
